# Application of Metal-Based Nanomaterials in In Vitro Diagnosis of Tumor Markers: Summary and Prospect

**DOI:** 10.3390/molecules28114370

**Published:** 2023-05-26

**Authors:** Xiaobo Yang, Shaodian Zhang, Nong Lin

**Affiliations:** 1 Department of Orthopedic Surgery, The Second Affiliated Hospital, Zhejiang University School of Medicine, Hangzhou 310019, China; yangxiaobo20@zju.edu.cn (X.Y.); 12218598@zju.edu.cn (S.Z.); 2 Orthopedics Research Institute of Zhejiang University, Hangzhou 310009, China; 3 Key Laboratory of Motor System Disease Research and Precision Therapy of Zhejiang Province, Hangzhou 310009, China; 4 Clinical Research Center of Motor System Disease of Zhejiang Province, Hangzhou 310009, China

**Keywords:** metal-based nanoparticles, cancer, in vitro, early diagnosis

## Abstract

Cancer, which presents with high incidence and mortality rates, has become a significant health threat worldwide. However, there is currently no effective solution for rapid screening and high-quality treatment of early-stage cancer patients. Metal-based nanoparticles (MNPs), as a new type of compound with stable properties, convenient synthesis, high efficiency, and few adverse reactions, have become highly competitive tools for early cancer diagnosis. Nevertheless, challenges such as the difference between the microenvironment of detected markers and the real-life body fluids remain in achieving widespread clinical application of MNPs. This review provides a comprehensive review of the research progress made in the field of in vitro cancer diagnosis using metal-based nanoparticles. By delving into the characteristics and advantages of these materials, this paper aims to inspire and guide researchers towards fully exploiting the potential of metal-based nanoparticles in the early diagnosis and treatment of cancer.

## 1. Introduction

Cancer is a prevalent and lethal disease that constitutes a significant public health concern on a global scale. In China, the prevalence and mortality of cancer are higher than most countries in the world, and these data are showing a rapid elevated trend [1,2,3,4]. The uncontrolled proliferation of cancer cells, often accompanied by infiltration and metastasis, contributes significantly to the elevated mortality rate [5,6]. The development of early-stage cancer diagnosis and clinical targeted therapy has thus become a focal point of research. Conventional techniques, such as cytological examination, imaging examination, and immunohistochemical examination, are frequently employed for the detection and identification of malignant tumors. While imaging technologies such as CT, MRI, and PET-CT can accurately locate the size, shape, and location of tumors, early-stage lesions of certain cancers may be challenging to detect. Moreover, the use of radiation or contrast agents carries inherent risks of toxicity and irradiation. Although cytological examination can detect cell morphology and structure and provide quick diagnosis for certain cancers, it is susceptible to misjudgments of normal cells as cancer cells or vice versa. Immunohistochemical examination, which detects specific proteins in tissue to determine the type and classification of cancer cells, offers a more accurate diagnosis and personalized treatment plans; however, this method typically requires invasive sampling, which may pose unpredictable risks, such as bleeding or infection.

As an emerging technological modality, nanotechnologies have demonstrated notable advantages and extensive potential for application in the field of biomedicine. At present, nanotechnologies have been widely used in the diagnosis and treatment of diseases, such as drug delivery [7,8,9], targeted therapy [10,11], detection and diagnosis [12], and molecular imaging [13,14]. Metal-based nanoparticles (MNPs) are a kind of nanomaterials with extensive and meaningful application possibilities, which can exert a variety of unique physical, chemical, and biological properties and then show strong potential in the early diagnosis of cancer. The principal detection mechanism of this method is grounded on the surface plasma resonance enhancement effect and local surface plasma resonance effect of nanomaterials, as well as their distinct optical, electrical, thermal, chemical, and other properties, which are leveraged through specific biomarker detection methodologies. At present, MNPs have made great strides in in vitro and in vivo screening technologies for early-stage tumor diagnosis. As an illustration, magnetic nanoparticles can be employed in MRI and magnetic navigation technology to enhance the precision and accuracy of cancer cell and metastasis localization and sizing.

As the latest review of recent studies on the use of MNPs, the present review provides a comprehensive summary of the recent research progress in the development of tumor marker biosensors utilizing MNPs. The review appropriately categorizes and summarizes the various types of MNP sensors, bifurcating them into two distinct research directions based on their catalytic and optical properties. Being different from previous studies, we also proposed the challenges of MNPs in clinical application and the main development directions in the future, hoping to provide scientific and valuable ideas for researchers, so that MNPs can give full play to their unique advantages and make contributions to the construction of biosensors with practical application value.

## 2. Introduction for Tumor Markers

MNPs, as a class of nanomaterials with robust catalytic activity, favorable electrical conductivity, and exceptional optical properties, among other physical and chemical attributes, represent a prime candidate for fabricating biosensors aimed at early cancer diagnosis via the detection of tumor markers. Tumor markers are a group of substances that are overexpressed by the body or cancerous cells during the onset and progression of cancer and play a significant role in the timely diagnosis of cancer patients. Some existing research reports have proved that biosensors constructed based on MNPs can be used for the detection of tumor markers.

**Figure 1:**
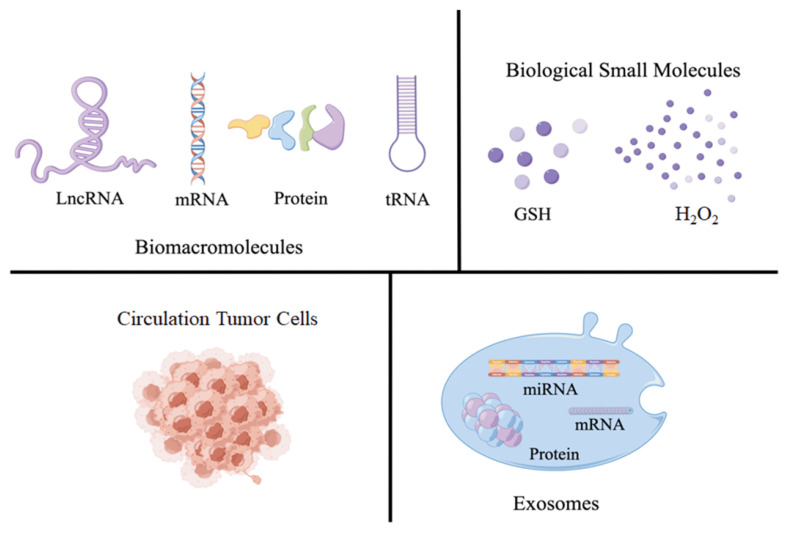
The main types of tumor markers. GSH: glutathione.

Based on published literature, tumor markers can be classified into four distinct groups: biomacromolecules, biomolecules, circulating tumor cells (CTC), and exosomes (Figure 1). Among which, biomacromolecules include nucleic acids and proteins. Different kinds of nucleic acids, such as mRNA, miRNA, and tRNA, play an important role in many pathophysiological phenomena and cell differentiation and proliferation processes, so they can be regarded as markers for early cancer diagnosis. On the other hand, proteins serve as the primary building blocks in the process of cellular proliferation, the expression of tumor-related proteins with explosive growth in early cancer patients, which enables it to accurately determine whether the patient is undergoing disease. For example, the corresponding miRNAs show different expressions in colorectal cancer, pituitary cancer, and other cancers [15,16,17]. Alpha fetoprotein (AFP) or cancer embryo antigen (CEA) can be used as characteristic markers in the early diagnosis of gastric cancer, lung cancer, breast cancer, etc. [18,19,20]. So far, there are more than 160 different types of tumor markers that can be used for the diagnosis of early-stage cancer patients, including hydrogen peroxide (H_2_O_2_), glutathione (GSH), and other biomolecule compounds [21,22,23], CTC [24], exosomes engaged in regulating cell waste disposal and intercellular communication, etc. [25].

## 3. The Catalytic Properties of MNPs for Detecting Tumor Markers

### 3.1. Construction of Electrocatalytic-Activity-Based Electrochemical Biosensor

In the biological analysis field, electrochemistry and its related technologies can be used as an effective biosensor tool to transform the target analyzer into an electrochemical signal that can be accurately measured [26]. Due to their large grain boundary ratio and high specific surface energy, MNPs can adsorb and bond with other substances, and are widely used in the modification of electrode materials [27,28,29]. At present, the improvement of the biosensor includes two directions: one is switch to a new signal amplification marker to enhance sensor functionality, and other is design and develop a substrate material with better biocompatibility and conductivity, thereby increasing the antibody load and speeding up electron transfer [30].

**Figure 2:**
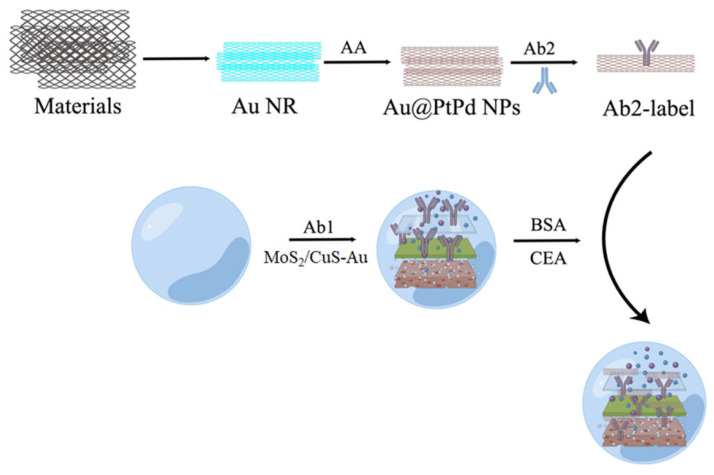
The construction of a MoS_2_/Cu-Au sensing platform and the inspection process of CEA: the proposed immunosensor was mainly synthesized by hydrothermal method, and antibodies (Ab2) are utilized to achieve accurate detection of CEA. CEA: cancer embryo antigen; BSA: bovine serum protein.

Using MoS_2_/Cu-Au as the sensing platform and Mulberry-like porous nanorods (Au@PtPd MPs) as the signal amplifier, Dong’s group successfully constructed a sandwich-type electrochemical immune sensor for the precise detection of CEA with high sensitivity (Figure 2) [31]. Through the test, it was found that the immunosensor showed a wide linear detection range of CEA (50 fg/mL~100 ng/mL), and the lowest limit of detection can reach 16.7 fg/mL. In the presence of interfering substances, such as prostate-specific antigen (PSA), human immunoglobulin G (HIgG), and AFP, the signal of CEA is significantly stronger than that of the interfering substance, and the interfering substance basically has no inductive signal. Upon comparison with the conventional enzyme-linked immunosorbent assay (ELISA) method, the study demonstrated that the variance between the two approaches was less than 5%, thereby verifying the linearity, specificity, and stability of the immunosensor output.

**Figure 3:**
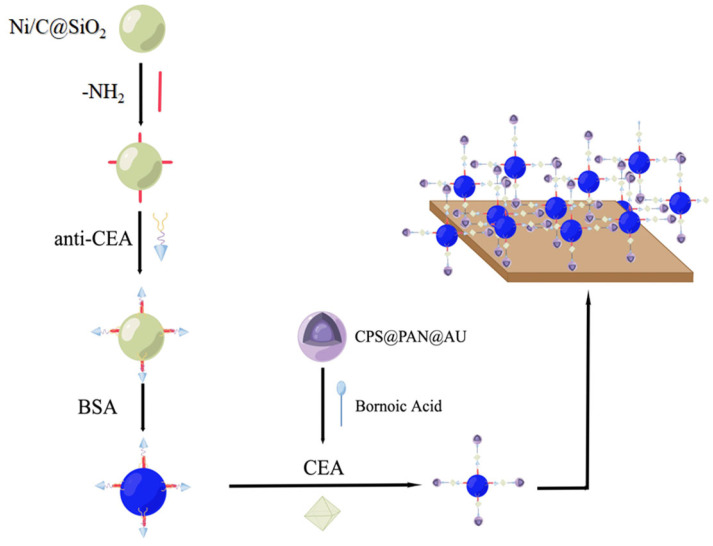
Scheme of the construction and detection principle of a sandwich-type electrochemical biosensor: the modification of materials was achieved in various solutions, including the introduction of amino and boronic acid groups. CEA: cancer embryo antigen; BSA: bovine serum protein.

Zhang et al., used hollow magnetic silica-coated nickel/carbon (Ni/C@SiO_2_) nanocomposites as the carrier and gold nanoparticle-coated polyaniline microspheres (CPS@PANI@Au) as the electrochemical biosensor to develop a sandwich-type electrochemical biosensor for CEA identification (Figure 3) [32]. A gold nanoparticle-coated PANI microsphere with good biocompatible properties can be used as a carrier for fixing proteins, and additional boric acid is introduced to fix the glycoproteins of CEA. On the one hand, hollow magnetic nanosilicon has hydroxyl groups that are easy to be modified on the surface, and on the other hand, it has a larger specific surface area, lower density, and simpler separation method. As a carrier, it has unique advantages for CEA detection with good selectivity and specificity. The complete distribution of PANI endows it with exceptional electron transfer properties, thereby enabling effective amplification of detection signals and leading to a substantial increase in the sensitivity of the sensor. By optimizing the test conditions, it was finally determined that the sensor showed excellent sensing performance. Compared with other interfering substances, such as bovine serum protein (BSA), lysozyme (Lys), and thrombin (Thr), CEA showed significant differences, and the lowest limit of detection was 1.56 pg/mL.

**Figure 4:**
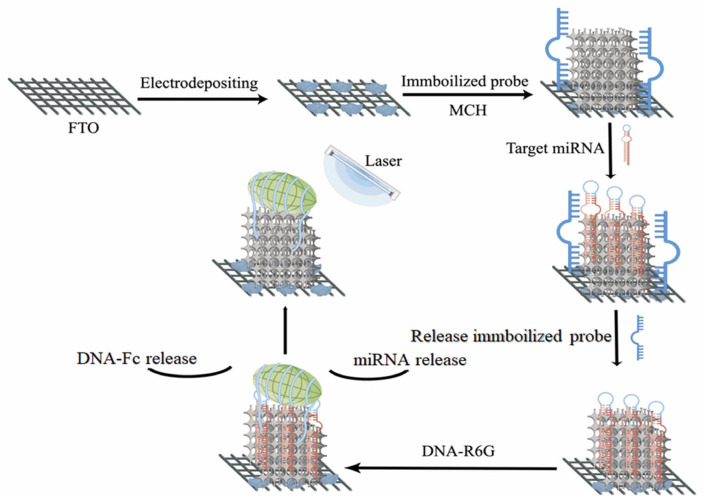
Molecular machine for miRNA detection by gold nanomembranes based on a 3D popcorn structure. FTO: fluorine-doped tin oxide coated glass; MCH: 6-mercapto-1-hexanol; DNA-R6G: DNA-rhodamine 6G.

miRNA is often considered unsuitable for use as a tumor marker due to its poor stability, complex structure, and difficulties in preserving and purifying samples, which result in nonspecific detection. In contrast, other markers, such as CEA and AFP, are more frequently used due to their ease of detection. Zhou et al., synthesized a gold nanofilm similar to a 3D popcorn structure as a new type of electrochemically active substrate, and constructed a biosensor, named “molecular machine”, for surface-enhanced Raman scattering (SERS) detection and electrochemical detection and miRNA (Figure 4) [33]. This approach significantly increases the number of active “hot spots,” thereby improving the sensitivity of SERS and electrochemical signals to exquisitely low detection limits, as low as 2.2 fM. The biosensor can also selectively monitor a variety of different biomarkers, making it an ultrasensitive miRNA detection platform by changing the corresponding probe sequence. The versatility and reliability of this method have been confirmed through its successful detection of miRNA levels in different cancer cells, indicating its promising potential for clinical applications. 

Before these biosensors mentioned above, many MNP-based electrochemical sensors were developed for the detection of cancer markers. These sensors have demonstrated practicality and reliability and possess a well-established detection system and mechanism. However, the metal elements used in these sensors are mostly precious metals, such as gold and platinum, leaving significant room for improvement in terms of material preparation and reusability (Table 1).

### 3.2. Chemical Catalysis-Based Tumor Marker Determination

The main principle of chemocatalytic determination of tumor markers is monitoring real-time color signals’ changes through colorimetric methods, and on-site analysis and instant diagnosis could be completed without complex and sophisticated equipment. At present, the reaction media used in this process are primarily synthesized using costly and potent natural enzymes. Nonetheless, the limited storage capacity and purification methods of natural enzymes have significantly constrained their widespread utilization [47]. MNPs have attracted the attention of researchers owing to their enzyme-like catalytic activity, and a series of colorimetric biosensors have been designed for testing and analysis of tumor markers.

Gao’s group had successfully integrated MoS_2_ nanosheets (MoS_2_ NSs) with peroxidase-like activity. Further, DNA modification was implemented to dramatically enhance the enzyme-like activity of the MoS_2_ NSs. Notably, the modified material exhibited a catalytic activity that was 4.3 times greater than that of unmodified MoS_2_ nanosheets. Based on the enhancement of DNA on the activity of MoS_2_ NS enzymes, a colorimetric sensing platform for the rapid and sensitive detection of CEA has been established [48]. The response results appeared linear across the range of CEA concentrations up to 1000 ng/mL, and the lower limit of detection can go down to 50 ng/mL. When there are interfering substances, such as AFP, mucin-1 (muc-1), and BSA, in the detection system, good specificity can be maintained. This work has represented a significant advancement in exploring the potential use of DNA as a modifier to enhance the enzyme catalysis of MNPs, and the findings have promising implications for the development of portable and visible tumor marker detectors.

Zheng et al., utilized the reduction and stabilization effects of NADH to synthesize ultrasmall Au–Pd nanoclusters [49]. The strong interaction between Au–Pd enhances the electron transfer of Au and Pd in bimetal nanoclusters, Au–Pd nanoclusters has better enzyme-like activity than monometal nanoclusters, and the reaction rate is more than 20 times that of monometal elements. Based on the acidic conditions required for the peroxidase-like activity of Au–Pd nanoclusters, a colorimetric method for the quantitative monitoring of acidic phosphatase (ACP) has been established, with a detection concentration range of 1–14 U/L, and the lower limit of detection is down to 0.53 U/L, and experiments have verified that this bimetallic catalytic enzyme has excellent sensitivity, selectivity, and recycling ability. This NADH-based nanoenzyme has shown promising universality and applicability in biological sensing, clinical diagnosis, etc.

**Figure 5:**
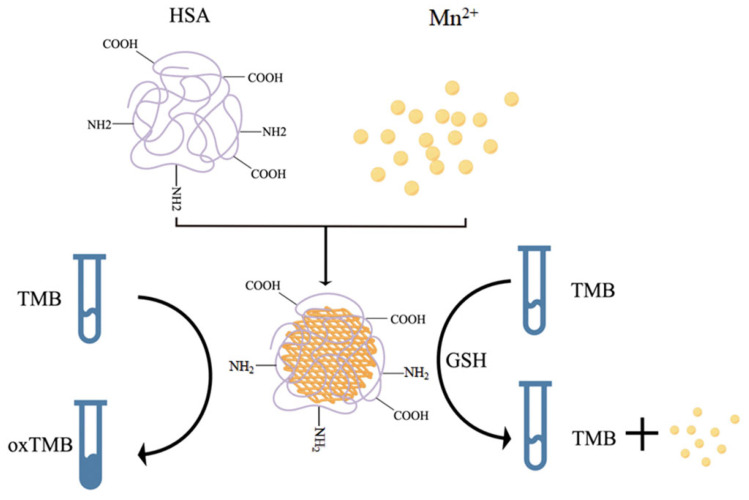
Scheme of detection of GSH in vitro based on MnO_2_ nanoplates: MNP material can interact with GSH to induce a color change of TMB, thereby achieving quantitative detection. HSA: human serum albumin; TMB: 3,3′, 5,5′-tetramethyl biphenylamine; GSH: glutathione.

In general, colorimetric analysis that relies on peroxidase enzymes typically involves the addition of H_2_O_2_, which often leads to toxic effects on cells. However, the use of MNPs as simulated enzymes eliminates the requirement for H_2_O_2_, greatly improving the accuracy of the test. Ge et al., developed a human serum albumin (HAS) template based on MnO_2_ nanosheet, which functions as an oxide analog enzyme for the colorimetric detection of the typical tumor marker GSH (Figure 5) [50]. The synthesized MnO_2_ nanosheet can directly catalyze the oxidation of 3,3′, 5,5′-tetramethyl biphenylamine (TMB) to produce blue ox-TMB. In the presence of GSH, the MnO_2_ nanosheets are reduced to Mn^2+^ due to the reaction with GSH, inhibiting the oxidation of TMB. As a result, the quantification of GSH is accomplished by detecting changes in absorbance that correspond with varying concentrations, thereby facilitating cancer diagnosis. Additionally, metal composite materials also exhibit good oxide analog enzyme activity. Guo et al., reported a new type of nanocomposite, namely, Cu^2+^-modified hexagonal boron nitride nanosheets loaded with gold nanoparticles (Au NPs/Cu^2+^-BNNS) [51]. The nanomaterial exhibits excellent oxide analog enzyme activity, which is derived from the adsorption of reactive oxygen species, and Cu^2+^ can synergistically promote the oxidation process. Notably, the color rendering of TMB induced by AuNPs/Cu^2+^-BNNS takes only 4 min, making it a useful tool for the rapid detection of CEA.

The detection of tumor markers using nanoenzymes has always been a key focus of academic research. While Table 2 highlights several noteworthy detection methodologies, it is worth noting that the literature cited in the table represents merely a small subset of the extensive reports available on this subject.

### 3.3. Methods-Based Photocatalytic Properties for Tumor Marker Detection

Aside from the aforementioned chemocatalytic and electrocatalytic activities, nanomaterials that exhibit photocatalytic properties can generate photoinduced holes under illumination, which can subsequently oxidize TMB, resulting in the production of a blue solution. Ding et al., developed the GO-C_3_N_4_-AgBr ternary heterojunction nano-photocatalyst, which facilitates the separation of photogenic electron–hole pairs under sunlight. The separated holes can sensitively catalyze colorless TMB to generate blue ox-TMB [64]. Next, PSA in the detection solution is collected based on the specific binding of antigen and antibody. Finally, TMB is added to the ABS buffer of the immune complex. Under visible light irradiation, a rapid color change is observed within 10 s, and the PSA level is quantitatively identified based on the color change. The results indicate that the limit of detection of PSA can be reduced to 20 pg/mL, and the sensitivity is adequate to monitor the amount of PSA in the serum in healthy individuals (which is around 4 ng/mL).

**Figure 6:**
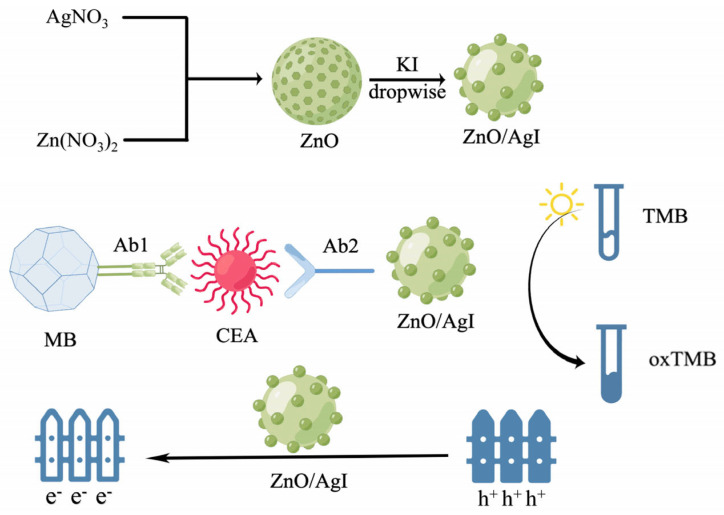
Scheme of the synthesis route of ternary ZnO/Ag nano-photocatalyst and colorimetric immunoassay for target CEA detection. CEA: cancer embryo antigen; MB: magnetic bead modified with -NH_2_; TMB: 3,3′, 5,5′-tetramethyl biphenylamine.

Zhang et al., utilized ZnO/AgI as a nanophotocatalyst for the in vitro detection of the tumor marker CEA (Figure 6) [65]. Under light irradiation, the ZnO/AgI nanomaterial undergoes electron transition to oxidize TMB. This nanomaterial is unique in that it possesses a suitable bandgap between ZnO and AgI, which facilitates electron–hole separation and enhances the efficiency of oxidation. The detection limit of ZnO/AgI for CEA can reach 65 pg/mL, and it maintains a good linear relationship within the concentration range of 0.1–7.0 ng/mL.

Employing the catalytic characteristics of MNPs for tumor marker detection represents an enhanced approach relative to conventional in vitro detection methods. This method relies on the catalytic impact of MNPs on biomarkers under specific conditions, thereby enabling the identification and diagnosis of cancer biomarkers. Constructing tumor marker sensors using the catalytic properties of MNPs is a widely researched and theoretically deep method. On the one hand, this category of methods is based on traditional detection methods, and on the other hand, the high catalytic performance can significantly convert and amplify biological signals, making MNPs suitable for detecting tumor markers. Building tumor marker sensors using electrocatalysis or chemical catalysis has shown good detection ranges and lower limits in single laboratory tests. However, it is important to note that the microenvironment in biological fluids is complex and flexible, and the stability, sensitivity, and specificity of sensors constructed based on the catalytic properties of MNPs to maintain excellent detection performance in the presence of various interfering substances are uncertain, which limits their ability to be transformed into practical applications. Building sensors using photocatalysis is a novel application direction for MNPs, but there is relatively little research on this topic. Although photocatalysis can quickly and accurately detect specific biomarkers, the essence is still based on colorimetric analysis. Although it is simple to operate, the reuse of the sensor remains insufficient. Overall, whether using electrocatalysis, chemical catalysis, or photocatalysis, they all exhibit advantages, such as high sensitivity, strong specificity, and fast reaction speed in the detection of tumor markers. However, their limitations include high cost, limited application range, and the impact of complex biological microenvironments on catalytic efficiency, which should not be ignored.

## 4. The Optical Properties of MNPs for Detecting Tumor Markers

### 4.1. Construction of MNPs’ Fluorescence Biosensor

Fluorescence-based sensors have high sensitivity and selectivity, which allows them a strong precedence in detecting tumor biomarkers. Due to their exceptional electronic structure and diminutive size, some MNPs commonly demonstrate high fluorescence quantum yields and molar extinction coefficients, along with notable photostability, making them frequently used for constructing fluorescence sensors. Quantum dots are one of the earliest nanomaterials applied in the field of bioscience. Compared with traditional organic dyes, they have higher quantum yields, better photostability, and longer luminescence lifetimes, but their toxicity and broad absorption band severely limit their use in biomedicine. In 2012, Luo et al., first discovered that the interaction between thiol salts and Au elements significantly amplified the intensity of luminescence in the materials. Subsequent studies revealed that the luminescence mechanism is achieved through ligand–metal charge transfer or ligand–metal–metal charge transfer, followed by radiation relaxation through a metal-centered triplet state, resulting in luminescence [66,67,68].

Huang et al., designed satellite structures of Au nanorods (Au NRs) and Ag_2_S quantum dots (Ag_2_S QDs) that are precisely regulated by DNA. The optimal metal-enhanced fluorescence effect is achieved when the distance between the two is approximately 8 nm [69]. In light of this, the structure is used for the accurate detection of the prostate cancer marker urine prostate cancer antigen 3 (PCA3), exhibiting high sensitivity and selectivity. The linear correlation between the variables is well suited within an interval of 5–500 pM, and the limit of detection for PCA3 is 1.42 pM. Importantly, the designed metal-enhanced near-infrared fluorescent satellite structure probe has been successfully used to detect PCA3 in human serum samples and prostate cancer cell lysates, demonstrating promising potential in the field of clinical cancer diagnosis.

CTCs are a significant cause of postoperative recurrence and distant metastasis in patients, as well as a critical factor leading to their mortality [70]. Many fluorescent nanomaterials emitting visible light have been applied to the subsequent detection of CTCs. Chen et al., proposed a sensitive, simple, and low-cost CTC detection strategy based on the selective recognition of Ag^+^ and C-Ag^+^-C by CdTe quantum dots, using muc-1 as the CTC marker and aptamers as the recognition probes [71]. The detection limit for muc-1 and A549 cells was 0.15 fg/mL and 3 cell/mL, respectively, and muc-1 at a mass concentration of 1 fg/mL and A549 cells at a concentration of 100 cell/mL could be visually distinguished. Yu et al., connected graphite carbon nitride quantum dots and gold nanocluster complexes with anti-EpCAM antibodies to obtain a CTC-specific ratio fluorescent immunoprobe, which can effectively capture and accurately quantify CTCs [72].

With improvements to MNP materials, they can currently be applied to detect tumor markers, such as ctDNA, exosomes, AFP, and PSA. Table 3 lists some MNP fluorescent detection methods used for tumor detection.

### 4.2. Build a Surface-Enhanced Raman Sensor

Surface-enhanced Raman scattering (SERS) is a fingerprinting technology that can reflect the vibrational characteristics of substances at the molecular level [87]. Compared with single-molecule detection methods, such as fluorescence spectroscopy, Raman spectroscopy-based methods have higher specificity. Distinct Raman spectra are exhibited by various tissues, cells, and bodily fluids within the human body. During the process of carcinogenesis, the configuration, conformation, and composition ratio of various biomolecules change, which may not cause clinical symptoms, but their Raman spectra will change. Therefore, SERS, with its high specificity, sensitivity, speed, and trace analysis capabilities, has gained increasing attention in the field of cancer diagnosis (Table 4).

MNPs are typically utilized as active substrates to enhance SERS signals, and the material type, shape, and size of the MNPs, as well as the adsorption amount and distance of probes on the active substrate, can impact the enhancement effect of SERS. The most commonly used MNPs are Au, Ag, and Cu [88,89]. Other transition metals or noble metals, such as Ni, Pt, and Pd, can also produce enhancement effects [90], but the effect is relatively low. Oxide semiconductors, such as TiO_2_ and ZnO, also exhibit a slight SERS enhancement effect [91]. For instance, Raymond et al., fabricated core–shell nanoparticles consisting of magnetic iron oxide and gold, and subsequently generated SERS nanotags in four distinct colors. They combined these with immunomagnetic separation to create a microfluidic device for capturing tumor cells bound to nanoparticles. They also quantitatively detected four surface protein markers of single tumor cells in whole blood. The sensor provides a straightforward and efficient platform for enhancing the accuracy of cancer metastasis observation and monitoring [92].

**Figure 7:**
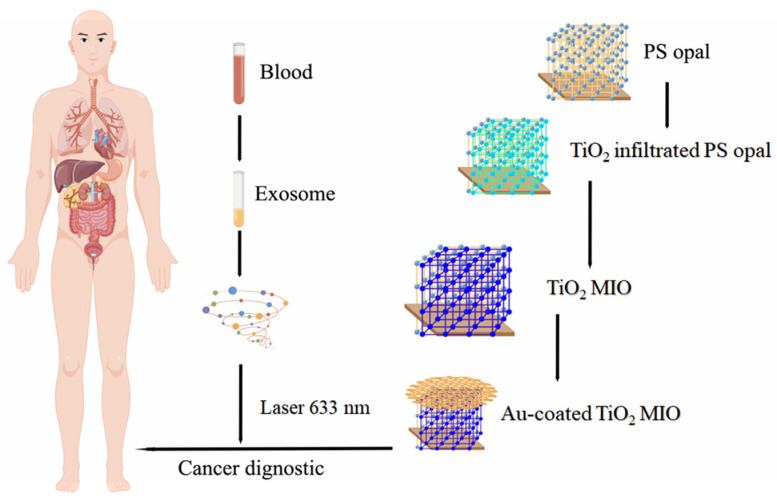
SERS inspection process of gold-coated TiO_2_. MIO: macroporous inverse opal; SERS: surface-enhanced Raman scattering.

Dong et al., developed a SERS-based nanoprobe with a gold-modified TiO_2_ large-pore inverse opal structure that exhibits a significant “slow light effect” and can be utilized for capturing and optically analyzing exosomes in plasma or other biological fluids (Figure 7) [93]. The probe demonstrated precise detection results for exosome detection in cancers, such as prostate, liver, and colon cancer, through the measurement of exosomes from cancer and normal cells in vitro.

**Table 4:** MNPs detection method based on SERS technology.

Materials	Tumor Marker	Linearity Range	Limit of Detection	Ref.
Small gold nanorods (Au NRs)	Exosomes of breast cancer cells	10^6^~10^8^ particles/mL	2 × 10^6^ particles/mL	[94]
Gold nanostar@4-mercaptobenzoic acid@nanoshell structures (AuNS@4-MBA@Au)	Exosomes of liver cancer patients	40~4.0 × 10^7^ particles/μL	27 particles/μL	[95]
Magnetic bead MB@SiO_2_@Au@aptamer	Exosomes of breast, colorectal, and prostate cancer	-	32, 73, and 203 particles/μL	[96]
Fe_3_O_4_@TiO_2_ nanoparticles	PD-L1 exosome	5 × 10^3^~2 × 10^5^ particles/mL	1 particles/μL	[97]
Fe_3_O_4_@Ag-DNA-Au@Ag@DTNB	miRNA	3 aM~100 pM	1.8 aM	[98]
Plasmonic head-flocked gold nanopillars@LNA detection probe	miRNA	1 aM~100 nM	1 aM	[99]
Functionalized gold nanoparticles (Au NPs)	muc-4	10 ng/mL~100 μg/mL	33 ng/mL	[100]
Silver/chitosan nanoparticles (Ag@CS NPs)	Platelet-derived growth factor BB	10 pg/mL~5.0 ng/mL	3.2 pg/mL	[101]
Fe_3_O_4_ nanoring (R-Fe_3_O_4_)	Interleukin-6	0.1~1000 pg/mL	0.028 pg/mL	[102]
4-MBA-encoded Au NPs (AuNP-MBA)	MCF-7	5~500 cells/mL	5 cells/mL	[103]
Triangular silver nanoprisms (Ag NPR)	HeLa cell	1–100 cells/mL	1 cell/mL	[104]
Poly(ethyleneimine) (PEI)-stabilized superparamagnetic iron oxide nanoparticles (SPION-PEI)	HeLa cell	1–25 cells/mL	1 cell/mL	[105]

### 4.3. Determination of Tumor Markers by Surface Plasmon Resonance Characteristics of MNPs

Nanoparticle aggregation can induce plasmon coupling between particles, resulting in a surface plasmon resonance (SPR) shift. Gold and silver nanoparticles (Au NPs, Ag NPs) are popular choices for colorimetric SPR detection among various nanomaterials. Cancer-related target molecules can trigger nanoparticle aggregation through covalent bonds, hydrogen bonds, hydrophobic forces, or electrostatic interactions. To improve the specificity and sensitivity of nanoparticle binding to cancer-related target molecules, nanoparticles can be surface-modified with ligands, antibodies, or other target molecules [106].

Szymańska et al., developed a surface plasmon resonance (SPR) imaging sensor for detecting CA125/MUC16 [107]. Anti-MUC16 antibodies were immobilized on a gold chip using a cysteamine linker and covalently attached with EDS/NHS. The sensor exhibited a linear response range of 2.2~150 U/mL, good recovery rate, and precision. It was successfully used to determine the markers in the serum of ovarian cancer patients.

Wang et al., developed a sensor with dual Au NP-assisted signal amplification for the highly sensitive detection of exosomes [108]. The sensor achieved double nanoparticle amplification and improved sensitivity by controlling the attachment of Au NPs through electronic coupling between the gold and Au NPs, as well as the coupling effect in the plasmonic nanostructure. The detection limit was lowered to 5 × 10^3^ particles/mL. Nonspecific adsorption of Au NPs on the SPR chip surface was also suppressed by coating the gold film surface with 11-mercapto-1-undecanol (MCU), enabling the regeneration of the SPR sensor.

**Figure 8:**
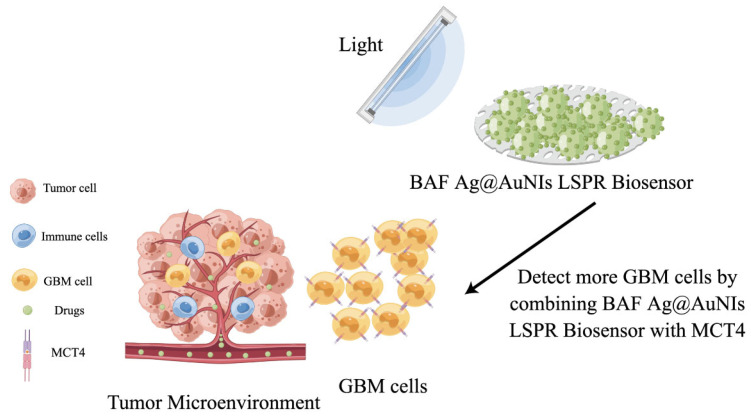
Scheme of GBM liquid biopsy using BAF Ag@AuNIs LSPR biosensor to detect enhanced MCT4 in blood-derived exosomes. GBM: glioblastoma; MCT4: monocarboxylate transporter 4; BAF: biotinylated antibody functionalization; LSPR: local surface plasmon resonance.

Liu et al., developed a local surface plasmon resonance (LSPR) sensor chip for the detection of exosomal biomarkers in small quantities (Figure 8) [109]. Self-assembled silver nanoparticles on a Ag@Au NIs sensor chip were utilized for the specific bio-binding of biotinylated antibodies, enabling the detection of exosomal surface biomarkers. The Ag@Au NIs LSPR biosensor with biotinylated antibody functionalization (BAF) sensitively detected CD63 (an exosomal biomarker) and monocarboxylate transporter 4 (MCT4) in malignant glioblastoma-derived exosomes, with a range of 4 × 10^−4^~50 μg/mL and a limit of detection of 0.4 ng/mL.

The use of MNPs’ optical properties for detecting cancer biomarkers is an emerging detection technology based on the principles of nanomaterial SERS and SPR. In specific tumor marker detection methods, the intensity and frequency of optical signals are observed to detect and diagnose cancer biomarkers with high sensitivity, improving the accuracy and reliability of early cancer detection. Additionally, MNPs’ surfaces can be recognized by specific biomolecules, enabling the selective detection of specific cancer biomarkers and reducing misdiagnosis rates. Compared with traditional cancer diagnosis techniques, the MNP-based optical detection of cancer biomarkers is relatively simple, requiring only simple chemical experiments and optical measurements. MNP preparation and biofunctionalization technologies are relatively mature, resulting in detection results with good repeatability and stability. However, its drawbacks are also apparent, as MNP preparation and functionalization are costly and time-consuming, leading to higher economic burden. The application of MNPs is limited by specific surface modification and functionalization, resulting in limitations in detecting certain types of tumor markers. MNP detection results require multiple experiments for verification to ensure accuracy and reliability. Overall, the use of MNPs’ optical properties for detecting cancer biomarkers has many advantages, but the application scope and reliability need further validation and exploration.

## 5. Conclusions and Prospect

Early and precise cancer diagnosis is a crucial factor in prolonging the survival rate of cancer patients and mitigating their suffering. Conventional methods for tumor biomarker detection are associated with limitations that include restricted sensitivity, potentially resulting in missed detection of early-stage cancer biomarkers, leading to incorrect diagnosis or delayed treatment. Furthermore, the low specificity of these methods means that certain tumor biomarkers may also appear in other diseases or healthy individuals, making it challenging to determine abnormal expression as a tumor biomarker. These methods also require a multitude of steps and complex operations, which increases the time and technical requirements and elevates the likelihood of misjudgment. Finally, the results may be impacted by various factors, such as disease, external interference, and physiological state, leading to ambiguous or unreliable outcomes. The development of nanotechnology has facilitated the extensive utilization of MNPs in diverse domains, encompassing biosensing and oncological interventions. This review focuses on the rapid development of MNP-based in vitro biosensors for cancer diagnosis in recent years and compares some promising technologies (Table 5).

MNPs are a new generation of biomaterials with broad application prospects and a profound impact compared with traditional early cancer diagnosis techniques. In the field of tumor biomarker detection, MNP-based biosensors demonstrate high sensitivity, rapid detection, good specificity, and ease of operation. However, the MNP-based nanomedicine field needs to address challenges and solve problems to achieve large-scale clinical applications. Primarily, although electrochemical sensors, chemical catalytic sensors, fluorescence sensors, and other sensor technologies based on MNPs demonstrate expansive detection capabilities and low detection thresholds for certain prototypical tumor biomarkers, it is imperative to acknowledge that the microenvironment of the biomarkers targeted by these sensors is relatively pristine and markedly distinct from that of physiological fluids. Therefore, the use of these sensors for early cancer diagnosis in actual cancer patients still has a long way to go, which is an important consideration for future researchers. Secondarily, the attainment of high specificity and sensitivity in detecting cancer-related tissues and organs through single biomarkers proves to be a complex task, thereby necessitating expeditious research into the physiological mechanisms of biomarkers, the exploration of novel biomarkers, and the integration of multiple biomarkers for clinical detection purposes. In the forthcoming times, scholars must prioritize enhancing the precision and sensitivity of biomarker detection, while concurrently monitoring market and application requirements, and devising compact, versatile, cost-effective, and expeditious platforms for biomarker detection. Thirdly, the current research on the intracellular and extracellular functional mechanisms of MNP sensors is severely insufficient. Hence, it is necessary to study the reaction mechanism of MNPs based on factors such as their chemical composition, size, shape, and synthesis method and apply the obtained rules to designing new biosensors, fully unlocking the potential of MNPs.

As a new generation of biomaterials, MNPs offer significant advantages over traditional tumor diagnosis technologies, such as high sensitivity, fast detection, good specificity, and simplicity. Nevertheless, challenges remain in achieving widespread clinical application. First, while MNP-based sensors provide a broad detection range and low limits for tumor markers, the microenvironment of detected markers differs from real-life body fluids, limiting early cancer diagnosis. Second, single biomarker detection is not sufficient, necessitating research on the physiological mechanism of markers, development of new markers, and multimarker clinical testing. In the future, biosensors will need to improve sensitivity and accuracy, address market needs, and develop a miniaturized, multifunctional, economical, and portable platform. Lastly, research on the functional mechanisms of MNP sensors is lacking, requiring the investigation of reaction mechanisms and the development of novel biosensors that maximize MNPs’ potential.

Currently, there are several drawbacks that hinder the further large-scale development of MNPs in the field of tumor biomarker detection, including high cost, limited selectivity, and poor biocompatibility. To address these issues, this paper suggests several improvements. First, enhancing the synthetic process of nanomaterials to streamline the procedure, amplifying the preparation efficacy, implementing integrated methodologies, exploring MNPs’ reuse and recovery, curtailing waste and environmental contamination, and diminishing production expenses. Second, researching new types of MNPs with improved selectivity for tumor biomarkers, reduced cross interference, increased biocompatibility, and safety. In this regard, it is possible to expand the application potential of inexpensive metals, such as iron, copper, and zinc, which are stable, safe, and nontoxic and easily undergo biological modification. Lastly, amalgamating MNP biosensors with other detection modalities, such as optical, electrochemical, and magnetic resonance techniques, can expedite the miniaturization and user-friendliness of MNP sensors, and heighten the precision and selectivity of tumor biomarker detection via the integration of diverse methodologies.

## Figures and Tables

**Table 1:** MNPs’ electrochemical sensor that has been reported in the literature.

Materials	Tumor Marker	Linearity Range	Limit of Detection	Ref.
N-doped graphene/Au nanoparticles (NG-AuNPs)	miRNA	10 fM~1 nM	0.17 fM	[34]
Multifunctional iron-based metal–organic frameworks (PdNPs@Fe-MOFs)	miRNA	0.01 fM~10 pM	0.003 fM	[35]
Unmodified gold nanoparticles (AuNPs)	miRNA	0.05~0.9 pM	16 fM	[36]
Gold nanoparticle (AuNP)-coated magnetic microbeads (AuNP-MMBs)	miRNA	5 fM~100 fM	0.14 fM	[37]
Graphite oxide–gold (GO-Au) nanocomposites	CEA	1~40 ng/mL	15.8 ng/mL	[38]
Silver nanoclusters (AgNCs@Apt@UiO-66)	CEA	0.01~10 ng/mL	0.3 ng/mL	[39]
Au@Ag nanoparticles (Au@Ag NPs)	CEA	0.0001~100 ng/mL	0.05 pg/mL	[40]
MoS_2_–Au composite–Ag NPs	CEA	1 pg/mL~50 ng/mL	0.27 pg/mL	[41]
Ag/MoS_2_@Fe_3_O_4_	CEA	0.0001~20 ng/mL	0.03 pg/mL	[42]
Platinum porous nanoparticles (Pt PNPs)	CEA, AFP	0.05 ng/mL~200 ng/mL	0.002, 0.05 ng/mL	[43]
Carbon–gold nanocomposite (CGN)	CEA, PSA, AFP	0.01~100 ng/mL	2.7, 4.8 and 3.1 pg/mL	[44]
Hollow gold nanospheres (HGN)	DNA	1~10 nM	1 pM	[45]
Magnetic mesoporous nanogold/thionine/NiCo_2_O_4_	SCCA	2.5 pg/mL~15 ng/mL	1.0 pg/mL	[46]

**Table 2:** MNP sensors based on chemical catalysis for marker detection.

Materials	Tumor Marker	Linearity Range	Limit of Detection	Ref.
Co/Mn oxide nanocomposite	ACP	0.02~1.0 U/L	8.2 mU/L	[52]
PdPt bimetallic alloy nanowires (Pd/Pt NWs)	ACP	0.17~2.67 U/L	0.06 U/L	[53]
Carboxylated chitosan modified Pt nanoclusters (CC-Pt NCs)	ACP	0.25~18 U/L	1.31 × 10^−3^ U/L	[54]
MnO_2_ nanosheets	ACP	0.075~0.45 mU/mL	0.046 mU/mL	[55]
Molybdenum oxide nanoparticles (MoO_3_ NPs)	ACP	0.09~7.3 U/L	0.011 U/L	[47]
CuO nanoparticles	cholesterol	0.625~12.5 μM	0.17 μM	[56]
Ultrasmall Pt nanoclusters (Pt NCs)	glucose	0~200 µM	0.28 µM	[57]
CuS nanoparticles (CuS NPs)	AFP	0.1–60 ng/mL	0.07 ng/mL	[58]
Two-dimensional Co_9_S_8_ nanocomposites (H_2_TCPP-Co_9_S_8_ nanocomposites)	H_2_O_2_	10~200 μM	8.19 μM	[59]
Porous 2D FeS_2_ nanosheets	H_2_O_2_	0.02~4.00 μM	7.60 nM	[60]
Pt–Ru bimetallic nanoclusters (Pt–Ru NCs)	H_2_O_2_	0.1~5 μg/mL	0.08 μg/mL	[61]
Dendritic mesoporous silica nanoparticle-MnO_2_	GSH	2~250 μM	0.654 μM	[62]
BSA-AuNP@ZnCo_2_O_4_ nanosheets	GSH	0.25~17.50 U/L	0.137 U/L	[63]

**Table 3:** Tumor marker detection scheme based on fluorescent sensor.

Materials	Tumor Marker	Linearity Range	Limit of Detection	Ref.
CuInSe2@ZnS nanoprobes	MCF-7 cells	10~5000 cell/well	12 cell/well	[73]
Functionalized Ag_2_S nanodot	MCF-7 cells	6–10 cell/mL	-	[74]
Pd nanosheets	ctDNA	1~100 nM	0.63 nM	[75]
Fe_3_O_4_ nanoparticles	ctDNA	100 amol/L~1 nmol/L	1.6 amol/L	[76]
Gold nanocages (Au NCs)	ctDNA	5 pmol/L~1000 pmol/L	6.30 pmol/L	[77]
Fe_3_O_4_ magnetic nanoparticle	Hepatic carcinoma-specific exosomes	576 (±15)~5.76 × 10^7^ (±5.1 × 10^5^) particles/mL	200 (±9) particles/mL	[78]
Ln-upconversion nanoparticles (UCNPs)	CEA	0.03~6 ng/mL	10.7 pg/mL	[79]
Carbon dots@SiO_2_ nanorods	CEA	1 fg/mL~10 ng/mL	794.6 ag/mL	[80]
Palladium nanoparticles (PdNPs)	AFP	5.0~150.0 ng/mL	1.38 ng/mL	[81]
Anti-AFP antibody functional gold nanoparticles (Au NPs)	AFP	0.50~45 ng/mL	400 pg/mL	[82]
ZnS nanospheres modified with CdTe quantum dots	AFP	0.04~64 ng·mL	10 pg/mL	[83]
NaYF^4^:Yb^3+^, Er^3+^@NaYF^4^:Yb^3+^ UCNPs	PSA	0.1~10 ng/mL	0.01 ng/mL	[84]
Fe_3_O_4_ magnetic-quantum dot nanobeads	PSA	0.01~100 ng mL	0.061 ng/mL	[85]
Entropy-driven amplification system-templated silver nanoclusters (Ag NCs)	miRNA	0~50 nM	8.7 pM	[86]

**Table 5:** Comparison of tumor marker detection techniques.

Technology	Basic Principle	Advantage	Disadvantage
Electrochemical sensors	Converts an interaction signal between a biometric element and a recognition target into a detectable electrical signal	High selectivity; mass production and integration; rapid and low cost; simple and suitable for complicated situation	Lack of specificity for the captured cancer cells; lack of ability to detect intracellular protein markers
Fluorescence	Change of fluorescence spectrum and fluorescence intensity	High selectivity and stability; simplicity and rapidity; good accuracy; biocompatible	Spectral overlap; photobleaching; nonspecific binding labeling
SERS	Difference in Raman scattering spectra of different molecule	High selectivity and sensitivity; noninvasive and nondestructive	Expensive and complicated equipment; batch-to-batch reproductivity of SERS substrate
SPR	Refractive index changes occurring from the capture of a molecule on the plasmonic surface	Real-time; free-label; high accuracy; suitable for different biofluids	Limited detection; interference from complex samples

## Data Availability

Not applicable.
